# Neurological manifestation of HEV infection: still a rare disease entity?

**DOI:** 10.1007/s00415-023-11985-8

**Published:** 2023-09-22

**Authors:** Maximilian Wiesenfarth, Thomas Stamminger, Eugen Zizer, Hayrettin Tumani, Albert C. Ludolph

**Affiliations:** 1 Department of Neurology, Ulm University, Oberer Eselsberg 45, 89081 Ulm, Germany; 2 Institute of Virology, Ulm University, 89081 Ulm, Germany; 3 Internal Medicine I, University Hospital Ulm, 89081 Ulm, Germany; 4 German Centre for Neurodegenerative Diseases (DZNE) Site Ulm, 89081 Ulm, Germany

**Keywords:** Hepatitis E virus, Neurological manifestation, Neuralgic amyotrophy, Plexus neuritis, Encephalitis, Guillain–Barré syndrome

## Abstract

Hepatitis E virus (HEV) infection is the most common form of viral hepatitis and is reported to cause neurological manifestation in up to 30% of diagnosed infections. We evaluated the medical reports of all patients (*n* = 29,994) who were discharged from the Department of Neurology of Ulm University between 01.01.2015 and 30.09.2022 to detect neurological manifestations of HEV*.* In addition, we retrospectively analyzed the serum samples of *n* = 99 patients representing different neurological diseases possibly related to HEV for anti-HEV-IgM and anti-HEV-IgG. At the time of discharge from hospital, the etiology of neurological symptoms in these patients was unclear. Overall, five cases of extrahepatic neurological manifestation of HEV (defined as anti-HEV-IgM and HEV-IgG positive) could be detected. An increase of both, anti-IgM- and anti-IgG-serum levels was significantly more common in neuralgic amyotrophy/plexus neuritis/radiculitis than in AIDP/CIDP (*P* = 0.01), meningitis/encephalitis (*P* = 0.02), idiopathic peripheral facial paralysis (*P* = 0.02) and tension headache (*P* = 0.02). In 15% (*n* = 15 out of 99) of retrospectively analyzed serum samples, conspicuous positive anti-HEV-IgG levels were detected. This finding was most common in AIDP/CIDP. In conclusion, results of this study indicate neurological manifestation of HEV to be a rare but still underestimated course of disease, occurring at any age and gender. Therefore, testing for HEV should be considered in patients with neurological symptoms of unknown origin, especially in those with neuralgic amyotrophy/plexus neuritis.

## Introduction

Hepatitis E virus (HEV) infection is caused by a RNA virus from the family of Hepeviridae, which can be differentiated into eight genotypes. In Europe and North America, infections are almost exclusively due to zoonotic transmission by genotypes 3 (and 4), which are endemic in these countries, mainly through insufficiently cooked pork and wild boar meat [1]. Fecal–oral transmission by genotypes 1 and 2 is rare and mainly detected in travelers returning from endemic areas in Asia and Africa [1, 2]. In recent years, the reported number of cases in Germany increased continuously until a slight decline in 2020 [3]. However, with an antibody prevalence of approx. 17% anti-HEV-IgG in Germany and 3246 infections reported to the Robert-Koch-Institute in 2020 [2–4], a large number of unreported cases can be assumed. The incidence of HEV infection was specified as 3.9/100,000 inhabitants in 2020 [3]. HEV infection is in most cases (about 95%) asymptomatic and even symptomatic patients mostly develop self-limiting symptoms such as mild gastrointestinal discomfort or general symptoms such as fever, tiredness and loss of appetite, but only rarely jaundice. In addition, there are also neurological, renal, hematological, cardiac and rheumatological symptoms described [1]. Neurological symptoms are reported in up to 30% of diagnosed infections and are therefore the most common extrahepatic manifestation of HEV [5]. A causal relation is particularly assumed in neuralgic amyotrophy (mostly bilateral) in approx. 10% [5–7], Guillain–Barré syndrome (GBS) in approx. 8% [8], myalgia in approx. 20% [5] and encephalitis/myelitis in case reports [1]. The pathomechanism of extrahepatic neurological manifestation of HEV infection is not yet completely understood. A direct neurotropic affection [1, 9] with viral replication in the central nervous system (CNS) [10] and an autoimmune reaction as a result of molecular mimicry [1] are discussed. The incubation period is about 15–64 days, although the beginning of the contagiousness has not been finally clarified. Diagnostic testing should include both anti-HEV-IgM (and anti-HEV-IgG) and HEV-RNA in serum or stool. HEV-RNA can be detected in stool about a week before and up to 4–6 weeks after the onset of jaundice [1, 2]. The hepatic manifestation of an acute HEV infection usually does not require antiviral therapy. However, in the case of a severe course of disease or pre-existing liver damage, the application of ribavirin can be discussed [1]. Hence, the pathomechanism remains unclear, and there is no specific treatment of HEV-associated neurological manifestations. In case of neuralgic amyotrophy caused by HEV, administration of cortisone and intravenous immunoglobulins is described to have a positive effect on the course of disease [5]. Except of case report series, no data on positive effects of treatment with apheresis are available [11, 12].

## Materials and methods

### Subjects

We evaluated the medical reports of all patients (*n* = 3) who were discharged from the Department of Neurology of Ulm University between 01.01.2015 and 30.09.2022 with the main or secondary diagnosis of acute HEV infection (ICD B17.2)*.* To collect more information on HEV in neurological patients and to uncover possible overlooked associations between HEV infection and neurological manifestations in some of the patients, we also evaluated the data of *n* = 99 patients with serum samples available in our biobank and with the diagnosis of neuralgic amyotrophy/plexus neuritis, meningitis, encephalitis, AIDP/CIDP, respectively, as well as idiopathic peripheral facial paralysis, which was described to be related to HEV in a number of cases [13], and tension headaches as a control. After an underlying differential diagnosis was ruled out by reviewing the examination results and discharge letters from all of the patients with available biosamples, we randomly selected *n* = 99 from the appropriate patients. A balanced gender and age distribution were ensured in the groups we investigated depending on the biomaterial that was available. Due to the low incidence of unclear neuralgic amyotrophy/plexus neuritis/radiculitis among inpatients and patients presenting to the emergency unit, in our study, despite of including all patients available, there was a smaller cohort (*n* = 5) in the according group. The CSF and serum samples were obtained as a part of regular clinical assessment during the hospital stay or in the emergency room. If necessary, all the patients who received a lumbar puncture were asked if they agree to store CSF and a blood sample in the biobank for research purpose.

All patients provided written informed consent prior to analysis of biosamples. The study was approved by the local institutional ethics committee of the University of Ulm (application number 20/10).

### Outcomes

The data of all patients (*n* = 99) were analyzed. Furthermore, a subgroup analysis was performed for the subgroups AIDP/CIDP (*n* = 28), neuralgic amyotrophy/plexus neuritis/radiculitis (*n* = 5), meningitis/encephalitis (*n* = 21), idiopathic peripheral facial paralysis (*n* = 25) and tension headache (*n* = 20). The data of the three patients with an acute HEV infection (ICD B17.2) were analyzed separately.

### Hepatitis E IgM/IgG analysis

The blood samples were tested at the Institute of Virology, Ulm University using the EuroImmun anti-hepatitis E virus ELISAs for IgG and IgM (Euroimmune, Lübeck, Germany) as specified by the manufacturer.

Anti-HEV-IgG-EIA levels and anti-HEV-IgM-EIA levels of < 0.8 were classified as negative, 0.8–1.09 as borderline and ≥ 1.1 as positive. As anti-HEV-IgM was described to show cross-reactivity with other pathogens, for example, cytomegalovirus (CMV) and Epstein–Barr virus (EBV) [14], an acute HEV infection was defined as detection of positive anti-HEV-IgM and anti-HEV-IgG or HEV-RNA (if available).

### Statistical analysis

For descriptive statistics, median (IQR) is given as appropriate. For group comparisons, the Chi-square test was applied for nominal variables. Unpaired Student’s* t*-test was used analyzing continuous variables. *P*-values were adjusted for multiple testing using the Bonferroni–Holm equation. A *P*-value of < 0.05 was regarded as statistically significant.

Statistical analyses and figures were performed using GraphPad Prism version 9.5.1 for Windows, GraphPad Software, San Diego, CA, USA, http://www.graphpad.com.

## Results

Between 01.01.2015 and 30.09.2022, three inpatients with an extrahepatic manifestation of acute HEV infection were diagnosed and treated in the Department of Neurology of Ulm University. One of the three patients presented with a symmetric neuritis of the brachial plexus, one patient with a predominant plexus neuritis/neuralgic amyotrophy of the left shoulder and one patient suffered from encephalitis. The medical history of these three patients is presented in detail below (Table 1).

**Table 1:** Demographic and clinical data of patients diagnosed with neurological manifestations of acute HEV infection

	Case 1	Case 2	Case 3
Age (years)	49	46	42
Sex	Female	Male	Male
Onset of symptoms	One day before admission	Hours before admission	Two months before admission
Symptoms	Word-finding difficulties, exhaustion, fever	Pain in both shoulders, weakness of the left shoulder	Painful weakness of upper extremities, especially left side
ALT (U/l) (ref. range: < 46)	139	406	212
AST (U/l) (ref. range: < 37)	102	91	71
GGT (U/l) (ref. range: < 55)	99	130	37
CSF leukocytes (…/µl) (ref. range: 0–4)	18	8	3
CSF total protein (mg/l) (ref. range: < 500)	956	620	686
Albumin CSF/serum ratio (… × 10^−3^) (ref. range: < 8)	15.2	8.8	6.8
CSF oligoclonal IgG bands pattern (Type 1–5)	Type 4	Type 5	Type 1
HEV serology	Anti-HEV-IgM, anti-HEV-IgG positive, HEV-RNA in serum positive	Anti-HEV-IgM, anti-HEV-IgG positive, HEV-RNA in serum positive	Anti-HEV-IgM, anti-HEV-IgG positive, HEV-RNA in serum negative
Diagnosis	Encephalitis	Neuralgic amyotrophy	Bilateral plexus neuritis
Therapy	Symptomatic therapy	500 mg methylprednisolone i.v. over 5 days	1000 mg methylprednisolone i.v. over 5 days

*ALT* alanine aminotransferase, *AST* aspartate aminotransferase, *GGT* gamma-glutamyl transferase, *HEV* hepatitis E virus

### Case 1

A 49-year-old patient was presented to the emergency unit because of acute word-finding difficulties that had occurred the day before. The patient felt exhausted for several days and an increased body temperature of 38.6 °C was measured. The physical neurological examination revealed no focal neurological deficit. The transaminases aspartate aminotransferase (AST) 102 U/l (< 32 U/l), alanine aminotransferase (ALT) 139 U/l (< 35 U/l) and gamma-glutamyl transferase (GGT) 99 U/l (< 38 U/l) were increased. In CSF, there was a pleocytosis of 18 leukocytes/µl (< 5/µl), increased total protein 956 mg/l (200–500 mg/l), a blood–CSF barrier dysfunction, i.e., albumin CSF/serum ratio = 15.2 × 10^−3^ (< 8 × 10^−3^) and identical oligoclonal bands in CSF and serum (type 4 pattern according to Andersson et al. 1994) [15]. Herpes simplex virus (HSV) or varicella zoster virus (VZV) infection were ruled out using antibody indices and PCR in CSF. The antibody indices for Borrelia and tick-borne encephalitis virus (TBEV) in CSF were also negative. Magnetic resonance imaging (MRI) of the neurocranium was normal. Electroencephalography (EEG) showed a slight diffuse brain dysfunction. Empirical antibiotic triple therapy with aciclovir, ampicillin and ceftriaxone was started. During the following days, a further increase in transaminases AST 275 U/l (< 32 U/l), ALT 442 U/l (< 35 U/l) and GGT 115 U/l (< 38 U/l) was observed. Abdominal sonography revealed normal findings. In a broad serological testing no evidence of an acute hepatitis A virus (HAV), hepatitis B virus (HBV), hepatitis C virus (HCV), CMV and EBV infection was found, however anti-HEV-IgM, anti-HEV-IgG and HEV-RNA in serum were positive. In conclusion, meningoencephalitis as an extrahepatic manifestation of acute HEV infection was diagnosed and antibiotic therapy was discontinued. Symptoms showed a complete regression.

### Case 2

A 46-year-old patient presented to the emergency unit with painful paresis of the left shoulder. He reported that he went to bed asymptomatically in the evening and woke up about two hours later with persistent, severe, stabbing pain in both shoulders. Taking ibuprofen provided only minor relief. In the morning, he noticed weakness in his left shoulder. The physical neurological examination showed paresis in the left arm and shoulder with deficits of abduction, external rotation and elevation without sensory deficits or reflex differences. The transaminases AST 91 U/l (< 37 U/l) and ALT 406 U/l (< 46 U/l) and the GGT 130 U/l (< 55 U/l) were elevated. In CSF there was mild pleocytosis of 8 leukocytes/µl (< 5/µl), increase of CSF total protein of 620 mg/l (200–500 mg/l), blood–CSF barrier dysfunction, i.e., albumin CSF/serum ratio = 8.8 × 10^−3^ (< 8 × 10^−3^) and monoclonal bands in CSF and serum (type 5 pattern according to Andersson et al. 1994) [15]. HSV or VZV infection was ruled out using antibody indices and PCR in CSF. MRI showed no evidence of cervical plexus affection and a normal neurocranium. In the nerve conduction studies of the median nerves, there was evidence of an early proximal lesion. In abdominal sonography, besides steatosis hepatis, there were normal findings. The hepatitis serology revealed an acute HEV infection with elevated anti-HEV-IgM 1.8 and anti-HEV-IgG 3.2, which was also confirmed by PCR. In summary, neuralgic shoulder amyotrophy as an extrahepatic manifestation of HEV was diagnosed. Therapy with 500 mg methylprednisolone i.v. was carried out over 5 days.

### Case 3

A 42-year-old patient, who was suspected to suffer from autoimmune polyneuritis, presented with a painful weakness of the upper extremities, in particular on the left side. Two months before admission, he noticed severe burning pain in both arms, in the back and on the medial side of the legs. Subsequently he developed a weakness in the arms, in particular on the left side. At the time of presentation, in the physical neurological examination an atrophy, hypaesthesia and allodynia in the left upper extremity, as well as paresis with reduced muscle reflexes in both arms were noted. The transaminases were slightly increased with AST 71 U/l (< 37 U/l) and ALT 212 U/l (< 46 U/l). In CSF there was a slight blood–CSF barrier dysfunction with albumin CSF/serum ratio = 6.8 × 10^−3^ (< 8 × 10^−3^) without evidence of pleocytosis and absence of oligoclonal bands (type 1 pattern according to Andersson et al. 1994) [15]. MRI of the spine showed normal findings apart from some intervertebral disc protrusions and extrusions. Electrophysiology was consistent with a left brachial plexus lesion. The serology showed positive anti-HEV-IgM 1.6 and anti-HEV-IgG 3.9. HEV-RNA was not detectable in the blood. An abdominal sonography was unremarkable. Assuming the diagnosis of bilateral plexus neuritis as an extrahepatic manifestation of an acute HEV infection, therapy with methylprednisolone 1000 mg i.v. over 5 days was started and then tapered off orally. In addition, neuropathic pain was treated with pregabalin and amitriptyline.

Overall, *n* = 99 patients were identified and included in the study for retrospective serological testing (Table 2). The sex ratio was almost balanced with *n* = 51 men and *n* = 48 women. Median age at the time of treatment in hospital was 52.0 years (IQR 35.0–65.5).

**Table 2:** Demographic data and serological findings of retrospectively analyzed neurological patients

	All patients	Neuralgic amyotrophy/plexus neuritis/radiculitis	AIDP/CIDP
*n*	99	5	28
Age (median, IQR)	52.0 (35.0–65.5)	61.0 (49.0–66.0)	66.0 (61.0–72.0)
Male	51.52% (*n* = 51)	20.0% (*n* = 1)	57.14% (*n* = 16)
Female	48.48% (*n* = 48)	80.0% (*n* = 4)	42.86% (*n* = 12)
Anti-HEV-IgM and -IgG increased	2.02% (*n* = 2)	40.0% (*n* = 2)	0% (*n* = 0)
Anti-HEV-IgG seroprevalence (borderline and positive)	15.15% (*n* = 15)	60.0% (*n* = 3)	25.0% (*n* = 7)

	Encephalitis/meningitis	Idiopathic peripheral facial paralysis	Tension headache
*n*	21	25	20
Age (median, IQR)	42.0 (37.0–69.0)	37.0 (30.0–53.0)	36.5 (28.0–52.75)
Male	42.86% (*n* = 9)	48.0% (*n* = 12)	50.0% (*n* = 10)
Female	57.14% (*n* = 12)	52.0% (*n* = 13)	50.0% (*n* = 10)
Anti-HEV-IgM and -IgG increased	0% (*n* = 0)	0% (*n* = 0)	0% (*n* = 0)
Anti-HEV-IgG seroprevalence (borderline and positive)	4.76% (*n* = 1)	8.0% (*n* = 2)	10.0% (*n* = 2)

*AIDP* acute inflammatory demyelinating polyneuropathy, *CIDP* chronic inflammatory demyelinating polyneuropathy, *HEV* hepatitis E virus

In 84% of the patients (*n* = 83) negative anti-HEV-IgG and anti-HEV-IgM titer in the serum were found. In 7% (*n* = 7) anti-HEV-IgG was positive and anti-HEV-IgM was negative, in an additional 6% (*n* = 6) anti-HEV-IgG was borderline. In two cases (2%) anti-HEV-IgG and anti-HEV-IgM were positive and in one case (1%) only anti-HEV-IgM was positive.

In the group of unclear neuralgic amyotrophy/plexus neuritis/radiculitis (*n* = 5), anti-HEV-IgM and anti-HEV-IgG was positive in two cases, whereas in one case only anti-HEV-IgG was elevated. Hence, an HEV infection as a probable cause of disease could be detected in 2/5 (40%) of patients with unclear neuralgic amyotrophy/plexus neuritis/radiculitis. These differences were statistically significant compared to AIDP/CIDP (*P* = 0.01), meningitis/encephalitis (*P* = 0.02), idiopathic peripheral facial paralysis (*P* = 0.02) and tension headache (*P* = 0.02).

In AIDP/CIDP patients (*n* = 28), anti-HEV-IgG was elevated in four and borderline in three patients. Conspicuous anti-HEV-IgG (borderline positive or positive) in 25.0% were more common compared to patients diagnosed with unclear meningitis/encephalitis (*n* = 1), idiopathic peripheral facial paralysis (*n* = 2) and tension headache (*n* = 2) overall (*P* = 0.02).

**Fig. 1:**
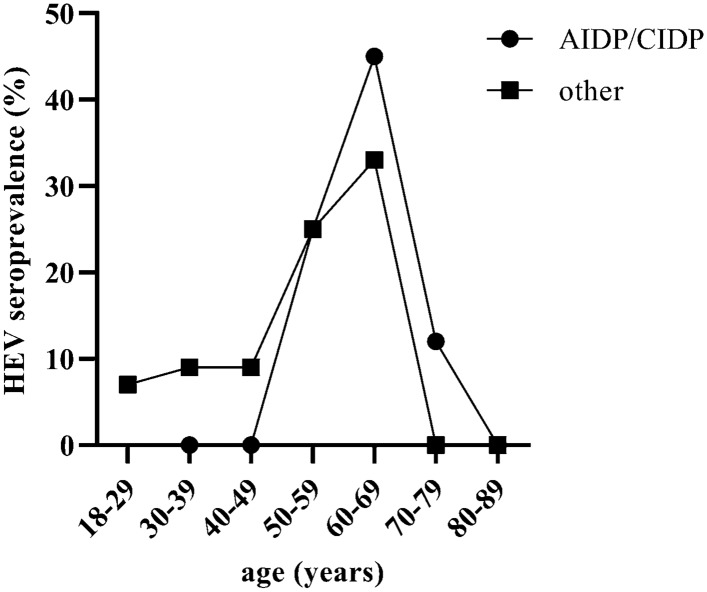
Comparison of anti-HEV-IgG seroprevalence (borderline and positive) in percent of patients with AIDP/CIDP (*n* = 28) and others (*n* = 71; plexus neuritis/radiculitis, encephalitis/meningitis, idiopathic peripheral facial paralysis and tension headache) by age. *AIDP* acute inflammatory demyelinating polyneuropathy, *CIDP* chronic inflammatory demyelinating polyneuropathy, *HEV* hepatitis E virus

Analyzing the results of anti-HEV-IgG serology by age, it was found that patients over the age of 50 years more often have a conspicuous anti-HEV-IgG serology (*P* = 0.02) (Fig. 1). The most common diagnosis in patients over 50 years of age was CIDP/AIDP. These patients showed a more frequent positive anti-HEV-IgG serology in relation to patients of the same age in the comparator groups. However, this was not statistically significant.

Compared to patients with negative HEV serology, liver enzymes (AST/ALT) in patients with elevated anti-HEV-IgM and/or anti-HEV-IgG were unremarkable and not significantly different.

When summarizing the three patients diagnosed with acute HEV in clinical routine and the two patients with retrospectively assessed increase of anti-HEV-IgM and anti-HEV-IgG in serological testing into one group, the median age is 46.0 (IQR 42.0–49.0). Three women and two men were affected. Four patients were diagnosed with neuralgic amyotrophy/plexus neuritis (one also affecting the lumbar plexus) and one patient with encephalitis.

**Fig. 2:**
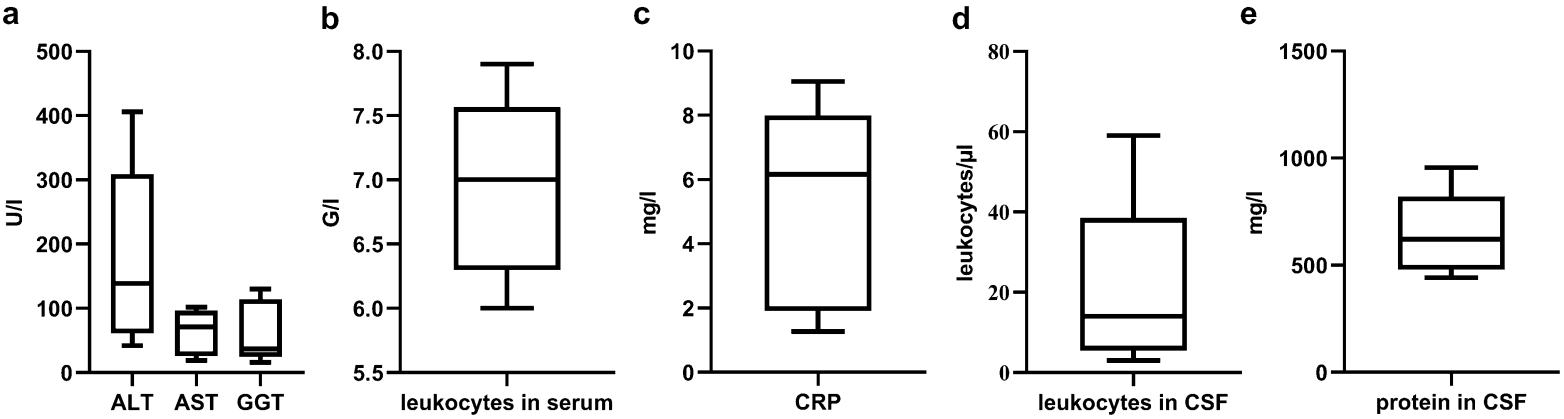
Laboratory parameters of patients with acute HEV infection (*n* = 5) in blood and CSF. Boxplots show median (IQR; minimum–maximum) **a** ALT, AST and GGT in U/l; **b** leukocytes in serum in g/l; **c** CRP in mg/l; **d** leukocytes/µl in CSF; **e** protein in CSF in mg/l. *ALT* alanine aminotransferase, *AST* aspartate aminotransferase, *GGT* gamma-glutamyl transferase, *CRP* c-reactive protein, *HEV* hepatitis E virus

Liver enzymes (especially ALT) were slightly elevated in four of five cases, while inflammation markers in blood (c-reactive protein (CRP), leukocytosis) were not detected. In CSF, a mild blood–CSF barrier dysfunction and/or pleocytosis could be observed in all patients suffering from extrahepatic manifestation of acute HEV infection (Fig. 2).

## Discussion

In our study, we screened a cross-section of patients with different neurological diseases for which an association with HEV infection is already discussed in the pre-existing literature. Therefore, we were able to record the frequency of patients with neurological manifestations presenting at a hospital of maximum care over a period of time of approximately 7.5 years. To uncover patients with a neurological manifestation of HEV that were missed in routine clinical practice, we performed a retrospective analysis of biosamples in *n* = 99 patients. In previous studies, HEV testing was mostly focused on specific entities of extrahepatic manifestation such as GBS [16], idiopathic peripheral facial paralysis [17] or acute infection of the central nervous system [18]. Likewise, in different countries serological testing for HEV was carried out in patients with a wide spectrum of non-traumatic neurological diseases [19, 20].

In our patients, five cases of extrahepatic neurological manifestation of HEV infection could be detected. The described disease entities neuralgic amyotrophy/plexus neuritis (*n* = 4) and encephalitis (*n* = 1) were consistent with previous reports in the literature, as these are reported to be the most common neurological manifestations of HEV [11, 19, 21, 22]. While it might not be surprising that two cases of HEV-associated neuralgic amyotrophy/plexus neuritis were missed in clinical routine, interestingly, these cases accounted for 40% of patients diagnosed with neuralgic amyotrophy/plexus neuritis. A relation between HEV and idiopathic peripheral facial paralysis could not be demonstrated in this study, as reported previously [17]. In line with the pre-existing literature, three of the four patients with neuralgic amyotrophy/plexus neuritis presented with bilateral affection [5]. The median age of the patients with neurological manifestations of acute HEV was 46.0 years (IQR 42.0–49.0) and, therefore, comparable to the literature [5, 11]. In contrast to the reported results [5, 11, 19], in our study more women were affected. However, this can be due to the small number of cases. As known from the literature, none of the patients were clinically jaundiced, and they presented at most only slightly increased liver enzymes [1, 19]. In accordance with existing guidelines of the underlying diseases the patients were mainly treated with corticosteroids and symptomatic analgesia. At this point in time, there is no causal therapy in neurological manifestations of HEV. For the treatment of the neurological syndrome with corticosteroids in patients with acute hepatitis E, positive effects have already been described previously and also most of our patients benefited from this therapy [5]. Nonetheless it is necessary to discuss the extent to which immunosuppression slows HEV clearance in these patients. The European Association for the Study of the Liver Clinical Practice Guidelines on hepatitis E virus infection recommends a corticosteroid therapy only in the case of acute liver failure [1]. Therefore, therapeutic strategies in patients with neurological manifestations of hepatitis E should be investigated in larger studies, including therapies such as apheresis and ribavirin, which are already used in severe cases [1]. A possible transmission route could be suggested in only one of the five patients by reporting the consumption of raw wild boar prior to the development of symptoms. A relevant history of travelling was not documented in any of the patients which tested positive, so transmission via food remains the most plausible cause. However, without sequencing of the genotype this hypothesis remains unclear. A sufficient cooking of meat and especially pork [23], which is one of the main transmission routes in western countries with reported seroprevalence in pigs of up to 90% [24], remains a good way to prevent infections.

Elevated anti-HEV-IgM and anti-HEV-IgG levels were found in 2% (2/99) of the retrospectively tested patients. This is comparable to the numbers of acute HEV described in neurological diseases such as GBS (1.2%) [16] and encephalitis (1.7%) [18], however, none of the GBS patients in our study presented with elevated anti-HEV-IgM. Of note, in the two retrospectively tested patients and in one of the patients diagnosed in routine clinical practice, an increase in anti-HEV-IgM and anti-HEV-IgG serum levels could not be confirmed by positive HEV-RNA in PCR. Nevertheless, in these cases we suspected a causal relationship between HEV infection and plexus neuritis due to significantly increased anti-HEV-IgM and anti-HEV-IgG serum levels, diagnosis-compatible clinical symptoms of unclear origin and normal findings in the extensive exclusion diagnostics. However, the causal relationship may be a post-acute infection syndrome (PAIS) rather than an acute infection. Neurological symptoms as a manifestation of PAIS exist in various viral infections and have recently been discussed extensively by Choutka et al. [25]. In the future, it will be interesting to evaluate the extent to which PAIS can cause neurological manifestations after HEV infection. According to different theories, PAIS can be induced by an immune reaction due to persistent viral infection undetectable by conventional methods, by an autoimmune reaction as a result of molecular mimicry, dysregulation of the virome, microbiome, mycobiome etc. or irreversible tissue damage during the acute infection [25].

In our study, elevated anti-HEV-IgM and anti-HEV-IgG serum levels were significantly more common in neuralgic amyotrophy/plexus neuritis/radiculitis than in AIDP/CIDP, meningitis/encephalitis, idiopathic peripheral facial paralysis and tension headache.

A multinational European study in the UK, France and the Netherlands found a comparable amount of acute HEV infection (2.4%) in neurologic patients. However, it must be differentiated in which of these patients the neurological disease is probably related to HEV infection and in which cases a random coincidence has to be discussed [19]. The influence of the patients’ disease spectrum on the obtained HEV seroprevalence has also to be discussed with regard to a large Chinese study reporting positive anti-HEV-IgM in only 0.54% of patients and 0.68% of controls, but in one of three GBS and two of 26 patients with viral encephalitis [20]. Moreover, a different influence of genotype 4, which is predominant in China, on the frequency and clinical phenotype of neurological manifestation of HEV must also be taken into account.

A higher rate of acute HEV (6.9%) in patients with acute neurological diseases was reported in a French study [22]. However, elevated anti-HEV-IgM in serum was considered to be sufficient for diagnosis in immunocompetent patients. The patients with positive anti-HEV-IgM mainly showed the typical neurological extrahepatic manifestations of HEV such as GBS, neuralgic amyotrophy and encephalitis [22].

Overall, when evaluating the relation of acute HEV and neurological diseases, differences in HEV seroprevalence must be taken into account, as regional hotspots are described [1]. Furthermore, it is believed that regional differences in clinical presentation as well as genotypes are relevant and should be considered when diagnosing neurological manifestations of HEV [26]. Due to the great heterogeneity of clinical phenotypes and the largely unknown pathomechanism, it is important to consider HEV in unusual neurological cases [27] in addition to the most common neurological manifestations GBS and neuralgic amyotrophy [6–8, 12, 16, 21, 28].

In contrast to the results of several studies from a neighbouring area in Germany, which reported an anti-HEV-IgG seroprevalence of 41% in GBS, 34% in facial paralysis or 30.7% in acute CNS infection [16–18], the anti-HEV-IgG seroprevalence of 15.2% (including borderline positive anti-HEV-IgG) in our study was clearly lower. Anti-HEV-IgG seroprevalence in our study was comparable to the overall prevalence in Germany [2–4]. Since the results mentioned above come from the same German federal state, it can be concluded that regional differences in HEV seroprevalence can be very pronounced even over short distances. Due to these differences in HEV seroprevalence, it may be useful to compare the frequencies of different neurological manifestations, as in our study, in patient groups with the same regional background, while the frequencies from different centers may be only partially comparable. However, in accordance to the previously reported results [16], we observed that the seroprevalence reaches a peak between 50 and 79 years.

In line with the previously reported unexpected high anti-HEV-IgG seroprevalence (41%) in GBS [16], we detected conspicuous anti-HEV-IgG being more common in patients with AIDP/CIDP (25%). A selection bias must be discussed, since the patients with AIDP/CIDP were older than in the comparator groups and the seroprevalence increases with age [16]. While there are large studies or case report series observing HEV infection and GBS, up to date there is little published data with regard to CIDP as a possible long-term result of HEV infection, for example, in the form of a PAIS. Considering the high amount of anti-HEV-IgG seroprevalence in our AIDP/CIDP group, as well as reported in GBS patients in Germany [16], it would be interesting to evaluate this topic in future studies.

Our study is not without limitations. It can be assumed that some of the noticed differences did not become statistically significant due to the limited number of cases, so that the study was underpowered. However, for most of the diagnoses, especially such as plexus neuritis/neuralgic amyotrophy, increasing the number of cases is difficult due to the low rate of inpatients treated. For this purpose, it would be necessary to carry out a multicentre study and to include outpatients with the diagnosis neuralgic amyotrophy/plexus neuritis. A strength of this study is the extensive availability of biomaterial from the biobank, enabling to retrospectively evaluate the serology of almost all treated patients in the last years with unclear AIDP/CIDP and neuralgic amyotrophy/plexus neuritis. Acquisition of HEV-RNA in blood or stool as well as genotype sequencing could secure the diagnosis and give more information concerning the route of transmission. Determining HEV-RNA and anti-HEV-IgM/anti-HEV-IgG from CSF would be necessary to evaluate suspected pathomechanisms. A detailed analysis of the discharge medical reports (including laboratory results from blood and CSF, MRI, electrophysiology etc.), ensured that only patients without any other differential diagnostic cause of the disease were included in all groups.

Results of this study show that neurological manifestation of HEV is a rare but still underestimated course of disease, occurring at any age and gender. Elevated liver enzymes and/or inflammation parameters can be indicative, but are not obligatory. Awareness of HEV is important in treating patients with neurological symptoms such as encephalitis, headache or peripheral nerve affection of unknown etiology. HEV serology should be routinely tested in neuralgic amyotrophy/plexus neuritis, as results of this study suggest a possible underlying causative relationship. High anti-HEV-IgG seroprevalence in AIDP and CIDP patients may indicate a possible relationship between past HEV infection and above mentioned clinical manifestations, however, this has to be re-evaluated in further studies including a larger number of cases.

## Data Availability

The data that support the findings of this study are available from the corresponding author, upon reasonable request.

## Abbreviations

AIDP: Acute inflammatory demyelinating polyneuropathy
ALT: Alanine aminotransferase
AST: Aspartate aminotransferase
CIDP: Chronic inflammatory demyelinating polyneuropathy
CMV: Cytomegalovirus
CNS: Central nervous system
CRP: C-reactive protein
EBV: Epstein–Barr virus
EEG: Electroencephalography
HAV: Hepatitis A virus
HBV: Hepatitis B virus
HCV: Hepatitis C virus
HEV: Hepatitis E virus
HSV: Herpes simplex virus
GBS: Guillain–Barré syndrome
GGT: Gamma-glutamyl transferase
MRI: Magnetic resonance imaging
PAIS: Post-acute infection syndrome
TBEV: Tick-borne encephalitis virus
VZV: Varicella zoster virus
